# Higher heritabilities for gait components than for overall gait scores may improve mobility in ducks

**DOI:** 10.1186/s12711-017-0317-2

**Published:** 2017-05-02

**Authors:** Brendan M. Duggan, Anne M. Rae, Dylan N. Clements, Paul M. Hocking

**Affiliations:** 1 The Roslin Institute and Royal (Dick) School of Veterinary Studies, University of Edinburgh, Easter Bush, Midlothian, EH25 9RG UK; 2 Cherry Valley Farms Ltd., Laceby Business Park, Grimsby Road, Laceby, North Lincolnshire DN37 7DP UK

## Abstract

**Background:**

Genetic progress in selection for greater body mass and meat yield in poultry has been associated with an increase in gait problems which are detrimental to productivity and welfare. The incidence of suboptimal gait in breeding flocks is controlled through the use of a visual gait score, which is a subjective assessment of walking ability of each bird. The subjective nature of the visual gait score has led to concerns over its effectiveness in reducing the incidence of suboptimal gait in poultry through breeding. The aims of this study were to assess the reliability of the current visual gait scoring system in ducks and to develop a more objective method to select for better gait.

**Results:**

Experienced gait scorers assessed short video clips of walking ducks to estimate the reliability of the current visual gait scoring system. Kendall’s coefficients of concordance between and within observers were estimated at 0.49 and 0.75, respectively. In order to develop a more objective scoring system, gait components were visually scored on more than 4000 pedigreed Pekin ducks and genetic parameters were estimated for these components. Gait components, which are a more objective measure, had heritabilities that were as good as, or better than, those of the overall visual gait score.

**Conclusions:**

Measurement of gait components is simpler and therefore more objective than the standard visual gait score. The recording of gait components can potentially be automated, which may increase accuracy further and may improve heritability estimates. Genetic correlations were generally low, which suggests that it is possible to use gait components to select for an overall improvement in both economic traits and gait as part of a balanced breeding programme.

## Background

Increases in growth rate and breast muscle mass which have been achieved through selective breeding of poultry have been associated with welfare problems, notably an increased incidence of poor gait (which includes ‘leg weakness’) [1–5]. Birds with leg weakness may suffer pain and have difficulty reaching food and water [1, 6–8], which lead to economic losses for the producer and possible starvation for the animals. Gait problems were first reported in turkeys and broiler chickens [9, 10], although early studies focussed mainly on the emergence of skeletal leg defects rather than gait itself [10, 11]. Poor gait has since been observed in other heavy meat-producing birds [3, 12–15]. Although in Pekin ducks poor gait has not been reported as extensively, there is concern that gait problems may appear in the future if selection for production traits continues along its current trajectory, mirroring their emergence in other poultry species. It is important to consider that while gait problems may be associated with pain, sub-optimal gait may also be simply a functional consequence of an altered morphology in lines which have been heavily selected for increased muscle mass [16, 17].

Traditionally, in chickens and ducks, poor gait is assessed and selected against by using a visual gait score [9, 18], which is an ordinal score given to each bird based on a visual assessment of how that individual walks. Although efforts have been made to refine the visual gait score [19], it remains a subjective measure of walking ability and thus is prone to error. Previous studies have found relatively moderate kappa coefficients (a measure of agreement between observers) between 0.6 and 0.8 in ducks and chickens [14, 20]. This may suffice for flock-level welfare assessments but is below the accuracy required for selection. An EU report on the welfare of broiler chickens acknowledges the subjective nature of the gait scoring system and highlights the need to develop a more objective system of assessing gait [21].

Gait is a complex trait that requires the integration of sensory input, balance, conformation and fine motor control, and heritability estimates for poultry gait tend to be low [22–24]. Similarly low heritability estimates have been published for visual gait scores in other species [25]. In addition, as the visual assessment of gait is a subjective measure [19], heritability estimates may be low, which limits potential genetic progress when selecting for such a trait. Attempts have been made to circumvent this problem of low heritability estimates by focusing selection on objectively measured traits such as tibial dyschondroplasia or bone deformity [26], although it remains unclear how these phenotypes affect the overall walking ability of birds. However, some gait components, such as step width, will certainly affect the overall walking ability of an animal and have not as yet been genetically evaluated.

The aim of this study was to estimate the reliability, heritability and genetic parameters of the visual gait score which is currently used in Pekin ducks and to compare this to heritability estimates for particular components of gait. It was hypothesised that these components of gait may be more heritable than the overall gait score. This was previously found to be the case in dairy cattle [25]. Components were chosen for ease of measurement as well as for their hypothesised influence on overall gait. This study focusses on two gait components: step width, which influences balance during the stride, and body roll, which is a proxy for medio-lateral centre of mass movement during walking. There may be other components of gait which are more central to the overall movement of the bird but we chose those due to their ease of measurement. The components were also chosen on the basis of our previous findings that poultry lines selected for breast muscle mass ambulate with a wider step width and at a slower velocity (which is likely to increase body roll for a given step width) [27]. The purpose of this study was to ascertain the suitability of selecting for gait components, rather than to identify which components, in particular, should become the focus of future selection programmes.

## Methods

### Assessment of gait score

In order to assess the reliability of the standard visual gait score in ducks, seven-week-old Pekin ducks were scored for gait by four industry gait scorers. Scorers were shown three video sequences of 36 birds walking over a runway. The video camera (Microsoft LifeCam Studio, recording at 30 frames per second) was placed behind each bird at a height of 15 cm. The video sequence contained 144 walks–four walks (including one duplicate) for each bird. Each walk lasted approximately 3 s in order to replicate the high throughput of birds during assessments on breeding farms. Scorers were asked to rate each walk with a score of 1 (very poor gait) to 5 (perfect gait). None of the scorers were informed that the sequences contained duplicate recordings or multiple walks from the same birds. Agreement between and within scorers was assessed by using Kendall’s coefficient of concordance and the Minitab software (Minitab version 17, Minitab Inc.).

### Measurement of gait components

Over the course of eight weeks, on one day per week, over 5000 Pekin ducks were visually scored for gait. On average, 650 birds were visually scored during each week. Two breeding lines (A and B) of Pekin duck were used, alternating each week. In total, data was collected from four hatches of each line (a different hatch was measured each week). These breeding lines are grandparent stock of the standard Cherry Valley commercial hybrid duck. Line A forms part of the maternal grandparent stock and Line B forms part of the paternal grandparent stock. All birds were hatched in the same hatchery and raised according to the Cherry Valley published guidelines. Water and feed (standard industry rations) were provided ad libitum. The photoperiod was 23 h light on day 1, reducing by 1 h per day until day 6 when the photoperiod was 18 h of light per day and this was maintained to the end of the trial. Phenotypic data collection for various traits was carried out on a breeding company farm by experienced members of staff. All phenotypic measurements took place at a single measurement station on the same breeding farm. After corralling birds at six weeks of age into a small area adjacent to the measurement station, each bird was weighed and its (ultrasonic) breast muscle depth was recorded. The birds were subsequently placed on a custom-built walkway (1.2-m wide and 4.8-m long) and allowed to walk away at their own pace, during which time each bird’s overall gait and gait components were scored (during normal selection procedures, birds are gait scored while walking over loose straw bedding). The walkway consisted of a wooden base (6-mm thick plywood) which was covered by a sheet of 7-mm green artificial turf in order to provide grip and to create a contrast so as to make the birds’ feet easier to see. Perspex sheeting (30-cm high) was fixed to the sides of the walkway to ensure that the birds walked straight to the end of the walkway. Gait was assessed using a visual gait score (which forms part of the company’s routine phenotypic measurement). Gait scores for Lines A and B were recorded by two different members of staff (each line was scored by only one individual), both of whom were experienced at scoring gait. The visual gait score used by the breeding company spans a 1 to 5 scale, with 1 representing a bird which is markedly lame and 5 representing perfect gait. The score for a bird was downgraded if, when walking, that individual displayed bowed or splayed legs, medially or laterally rotated feet, or if the angle of the back to the floor was outside the 35 to 65 degree range. Birds which were lame, immobile or walked on their hocks were given a score of 1. Most ducks were assigned scores between 2 and 4 (in this trial, 1% were given a gait score of 1; 29% a score of 2; 61% a score of 3 and 8% a score of 4).

In addition to the overall visual gait score, two gait components (step width and body roll) were recorded simultaneously by one of the authors (BMD). The same author scored components of gait for both lines. Step width was scored visually as the estimated distance (perpendicular to the direction of travel) between the most posterior parts of the feet on a 1 to 3 scale, a score of 1 denoting the feet as being very close together (or overlapping) and a score of 3 denoting that the feet were widely spaced during walking. Body roll (also on a 1 to 3 scale) was recorded as the degree of rolling of the shoulders during walking. This was considered an approximation of medio-lateral centre of mass movement since the position of centre of mass was impossible to ascertain visually. This trait was deemed important because the degree of medio-lateral movement of the centre of mass can affect the birds’ balance. Birds which display greater variation in centre of mass position during walking may be at greater risk of stumbles or falls. A score of 1 represented very little rolling of the shoulders, whereas a score of 3 was given to birds which rolled their shoulders to a large degree while walking. The repeatability for both gait component scores was assessed in a small trial at the beginning of this study and deemed to be satisfactory, although no larger scale repeatability test using video was carried out as was the case for the standard gait score. Birds which moved too quickly or too slowly to reliably score gait components were treated as missing values. Hence, gait components were recorded on 4252 of the 5251 birds that were phenotypically measured (Table 2). Among the birds measured, 5% were given a step width score of 1, 79% were given a score of 2 and 16% were given a score of 3. For body roll, 9% were scored 1, 74% were given a score of 2 and 17% were given a score of 3. In addition to standard phenotypic measures of breast depth and body mass, feed conversion ratios (FCR) for each bird were calculated by automated measurement of each bird’s individual feed intake and body mass. Data collected at the phenotypic measurement station was collated with information of the FCR of each bird. The pedigree of all birds was known, stretching back 15 generations.

This study was approved by the Veterinary Ethical Review Committee at the University of Edinburgh.

### Genetic analysis

Variance components resulting from univariate and bivariate mixed models of restricted maximum likelihoods were used to estimate heritability of the visual gait score and the gait component scores as well as to calculate the genetic correlations between traits using ASReml (ASReml-W, version 3, VSN International Ltd.). Six traits were analysed using the following model, which included fixed effects of sex and hatch and random effects of animal, pen and the permanent environment effect of the dam. The model terms were:

$${\mathbf{y}} = {\mathbf{Xb}} + {\mathbf{Za}} + {\mathbf{Vp}} + {\mathbf{Wd}} + {\mathbf{e}},$$

where ${\mathbf{y}}$ is the vector of trait measurements, ${\mathbf{b}}$ is a vector of the fixed effects accounting for the interaction between the hatch and the sex of each bird, ${\mathbf{a}}$ the vector of additive genetic effects, ${\mathbf{p}}$ is a vector of the pen effects, ${\mathbf{d}}$ is the vector of permanent environmental effects of the dam and ${\mathbf{e}}$ is the vector of residuals. ${\mathbf{X}}$, ${\mathbf{Z}}$, ${\mathbf{V}}$ and ${\mathbf{W}}$ are incidence matrices which relate the vectors ${\mathbf{b}}$, ${\mathbf{a}}$, ${\mathbf{p}}$ and ${\mathbf{d}}$ with ${\mathbf{y}}$. The variance/covariance structure was assumed to be:

$${\text{V}}\left[ {\begin{array}{*{20}c} {\mathbf{a}} \\ {\mathbf{p}} \\ {\mathbf{d}} \\ {\mathbf{e}} \\ \end{array} } \right]\left[ {\begin{array}{*{20}c} {{\mathbf{A}} \otimes {\mathbf{G}}} & {\mathbf{0}} & {\mathbf{0}} & {\mathbf{0}} \\ {\mathbf{0}} & {{\mathbf{I}} \otimes {\mathbf{P}}} & {\mathbf{0}} & {\mathbf{0}} \\ {\mathbf{0}} & {\mathbf{0}} & {{\mathbf{I}} \otimes {\mathbf{C}}} & {\mathbf{0}} \\ {\mathbf{0}} & {\mathbf{0}} & {\mathbf{0}} & {{\mathbf{I}} \otimes {\mathbf{R}}} \\ \end{array} } \right],$$

where ${\mathbf{A}}$ and ${\mathbf{I}}$ are the additive genetic relationship matrix and identity matrix, respectively. ${\mathbf{G}}$, ${\mathbf{P}}$, ${\mathbf{C}}$ and ${\mathbf{R}}$ represent the variance–covariance matrices of additive genetic effects, pen effects, permanent environmental effects of the dam and residual effects, respectively. A multinomial qualifier (with link functions between the observed and underlying scale) was not used as part of the model due to issues with convergence, probably due to limitations of the data structure and size of categorical traits. Residuals for these traits were normally and independently distributed. The pedigree and data structures are summarized in Tables 1 and 2, respectively.

**Table 1:** Pedigree structure for Lines A and B

Line	Individuals in pedigree	Generations in pedigree	Sires	Sires of sires	Dams of sires	Dams	Sires of dams	Dams of sires
A	120,031	15	1078	364	577	4039	663	1418
B	81,765	15	1078	377	535	3622	699	1349

Figures represent numbers of individuals

**Table 2:** Means (and standard deviations) for all traits measured in Lines A and B

Line	Number of phenotyped males	Number of phenotyped females	Gait score	Step width	Body roll	Finish weight (g)	Breast depth (mm)	Test FCR
A	1375	1254	2.80 (0.66) [0]	2.13 (0.46) [229]	2.08 (0.53) [230]	3760 (290) [0]	152 (15.3) [0]	1.90 (0.17) [887]
B	1342	1280	2.70 (0.56) [1]	2.10 (0.43) [269]	2.06 (0.47) [271]	3362 (297) [0]	146 (16.7) [0]	2.02 (0.23) [69]

Phenotypes were recorded at six weeks of age. Standard deviations and numbers of missing values for each trait are presented in round and square parentheses, respectively

## Results

Kendall’s coefficient of concordance, calculated between four experienced observers who scored gait in short video clips, was equal to 0.49 (df = 132, p < 0.001). The Kendall’s coefficient of concordance within observers (scoring duplicate videos) was equal to 0.75 (df = 135, p < 0.001). No clear observer drift effect was detected, i.e. scorers deviated to a similar degree when scoring the first 60 walks compared to the last 60.

Heritability estimates with genetic and phenotypic correlations for Lines A and B are in Tables 3 and 4, respectively. The heritability estimates of the standard gait score were low and standard errors in the female line were high. Estimated heritabilities for body roll and gait score were similar whereas for step width they were higher. Estimated heritabilities for economic traits (finish weight, breast depth and FCR) were generally moderate.

**Table 3:** Heritability estimates and correlations for gait and other major economic traits in Line A

Trait	Gait score	Step width	Body roll	Finish weight	Breast depth	Test FCR
Gait score	***0.061 (0.055)***	−0.346 (0.202)	−0.690 (0.146)	−0.703 (0.373)	−0.374 (0.319)	0.095 (0.303)
Step width	−*0.162 (0.034)*	***0.238 (0.074)***	0.561 (0.227)	0.217 (0.167)	0.066 (0.165)	−0.111 (0.181)
Body roll	−*0.337 (0.025)*	*0.282 (0.029)*	***0.079 (0.034)***	0.160 (0.215)	−0.033 (0.222)	−0.379 (0.218)
Finish weight	−*0.039 (0.030)*	*0.069 (0.034)*	*0.020 (0.029)*	***0.274 (0.091)***	0.452 (0.145)	0.609 (0.135)
Breast depth	*0.056 (0.040)*	*0.092 (0.028)*	*0.065 (0.037)*	*0.439 (0.028)*	***0.15 (0.074)***	0.205 (0.172)
FCR	*0.1226 (0.037)*	*0.007 (0.036)*	−*0.037 (0.032)*	*0.067 (0.036)*	*0.079 (0.031)*	***0.272 (0.096)***

Heritability estimates are in bold italics; genetic correlations are listed above the diagonal and phenotypic correlations are in italics, below the diagonal. Standard errors for all estimates are in parentheses

**Table 4:** Heritability estimates and correlations for gait and other major economic traits in Line B

Trait	Gait score	Step width	Body roll	Finish weight	Breast depth	Test FCR
Gait score	***0.115 (0.058)***	0.138 (0.199)	−0.506 (0.170)	0.126 (0.176)	−0.022 (0.186)	0.442 (0.136)
Step width	−*0.016 (0.028)*	***0.166 (0.058)***	0.571 (0.155)	0.029 (0.150)	−0.326 (0.151)	−0.156 (0.160)
Body roll	−*0.156 (0.028)*	*0.314 (0.023)*	***0.112 (0.047)***	−0.164 (0.163)	0.059 (0.173)	−0.136 (0.175)
Finish weight	*0.186 (0.025)*	*0.048 (0.027)*	*0.010 (0.025)*	***0.401 (0.090)***	0.230 (0.112)	0.303 (0.127)
Breast depth	*0.074 (0.024)*	−*0.034 (0.025)*	*0.046 (0.024)*	*0.390 (0.024)*	***0.295 (0.046)***	0.077 (0.140)
FCR	*0.071 (0.026)*	−*0.016 (0.027)*	*0.004 (0.026)*	−*0.079 (0.028)*	−*0.074 (0.025)*	***0.294 (0.048)***

Heritability estimates are in bold italics; genetic correlations are listed above the diagonal and phenotypic correlations are in italics, below the diagonal. Standard errors for all estimates are in parentheses

Phenotypic correlations between traits varied between lines. Generally, phenotypic correlations between gait traits and economic traits were very low and correlations between economic traits were also low, with the exception of finish weight and breast depth (Tables 3 and 4). Since in this study, relatively small sample sizes were used, estimates of genetic correlations between traits were associated with relatively high standard errors. Most genetic correlations between gait traits and economic traits were not significant (p > 0.05), with the exception of Line B, where significant genetic correlations were observed between step width and breast depth (t = 2.16, p < 0.05), and between gait score and FCR (t = 3.26, p < 0.01). The standard gait score had moderate to good genetic correlations with body roll (−0.51 to −0.69). Genetic correlations between gait score and step width were not significant. The significant genetic correlations between economic traits were moderate (0.23 to 0.61).

## Discussion

Gait problems are a major animal welfare issue facing modern poultry in intensive production systems. Our results suggest that a more targeted approach to assessing gait by focussing on gait components has the potential to improve progress in selecting for better gait in breeding birds.

The pilot study that involved a limited amount of data suggests that the current visual gait scoring system, while showing some level of agreement between scorers, may not be optimal for long-term use in breeding programmes, but can be improved. The Kendall’s coefficient of concordance suggests that low concordance exists between scorers. Indeed, when scoring video clips of the same walks (using the standard visual gait score described above), all four scorers agreed 28% of the time and three of the four scorers agreed 74% of the time. Individual scorers failed to allocate the same score to two duplicate walks 26% of the time. Some of these inconsistencies may be due to the short duration of each video recording. Short recordings were chosen in order to replicate conditions during assessments on farm; however for certain birds on farm, the scorer will observe a walk for longer than 3 s before allocating a score for that bird. The viewing angle of the camera, which was chosen to give a clearer view of the birds’ gait, is also different from the viewpoint used when scoring during selection on farm, which is from a standing position.

The suboptimal reliability of the visual gait score that was recorded by using these video clips suggests that an alternate and more rigorous method of gait assessment is required to make progress on selection for optimal gait as weight increases. Previous work on gait in cows suggested that assessing components of gait may yield higher heritability estimates [25]. Certain gait components such as step width and the ratio of double to single support time are known to have changed to a similar extent in both ducks and chickens which have undergone selection for increased body weight and meat yield [27], and selection decisions based on these components may yield greater progress than the current subjective gait scoring system.

This study estimated genetic parameters for components of gait and compared these to those of the overall visual gait score. The heritability of step width was higher than that of the original gait score in both lines and standard errors were approximately the same for both estimates. This is to be expected since the gait score is a subjective measurement based on a visual assessment of overall body movements, without any tangible reference points, whereas step width is a simpler score based on only one aspect of foot placement and therefore one would expect this score to be more objective. In addition, the recorder that measures step width can make use of reference points on the ground to compare successive birds. The heritability of body roll was similar to that of the gait score, probably because unlike step width, the assessment of body roll is a more subjective assessment. Heritability estimates for other economic traits (finish weight, breast depth and FCR) were in the range expected, with some differences observed between lines. For example, the mean estimate for the heritability of body weight in this study (0.34) is in a similar range than the heritabilities of 0.28 to 0.45 which were estimated in recent poultry studies [24, 28–30]. Heritability estimates presented in this study were calculated from only one phenotyped generation; thus, it is expected that these heritabilities would be estimated with more accuracy if more generations had been phenotyped, as is the case within commercial breeding programmes.

Phenotypic correlations between the gait score and production traits were generally low, which suggests that the gait score is indeed a measure of gait, rather than a proxy measure of body mass or breast depth. Phenotypic correlations between the gait score and components of gait were low to moderate, whereas those between each component of gait were generally moderate. Due to the relatively small sample size, genetic correlations were generally associated with relatively large standard errors that were of a similar magnitude to the genetic correlation estimates. The notable exceptions were the genetic correlations between gait score and body roll and between step width and body roll in both Lines A and B. In Line B, breast depth (considered a proxy for pectoral muscle mass) was negatively correlated with step width; continued selection for greater breast depth may result in a narrower step width. The effect of this narrower step width on balance will depend on the degree to which the body’s centre of mass moves laterally during gait. Genetic correlations between gait components and production traits were generally low and the data suggest that selection for improved gait will not be compromised by negative responses in economic traits.

These data demonstrate that the visual assessment of gait components during selection is both feasible and yields promising heritability estimates. While some caution must be exercised when interpreting these results (given the presence of categorical traits in the model), the use of gait components holds promise for future progress in selection for improved gait in ducks; since they are simpler traits, the assessment of gait components can be automated, for example by using pressure sensing technology as in Duggan et al. [27]. Automation of measurement has the potential to bring about greater objectivity and to increase breeding success. However, it is important to note that although the gait components that are the focus of this paper can be measured satisfactorily and have reasonable heritability estimates, it is not yet known which components should be selected to improve gait. For example, it could be argued that a wide step width would be beneficial to a bird with large lateral displacement of the centre of mass, whereas a narrow step width would be beneficial to a bird with little lateral centre of mass movement. However, it is also difficult to differentiate cause and effect associations between step width and lateral body movement. A more thorough understanding of how gait components are integrated to perform overall locomotion is therefore necessary before recommendations can be made on which particular gait components should be used in breeding programmes. It is likely that most of the improvement will be achieved using a selection index which combines weighted measurement of various gait components. Indeed, current overall gait scoring methods use a combination of components, which are subconsciously weighted in different ways depending on the observers’ opinions of what optimal gait entails. By focussing only on the measurement of gait components, this differential weighting among observers can be avoided.

## Conclusions

Scoring overall gait visually is a subjective measure which generally generates low (but useable) heritabilities. We demonstrate that focussing on gait components, rather than overall gait, may result in heritability estimates that are equal to or higher than those of the conventional visual gait score in ducks. The benefit of using components of gait is that their measurement can be automated to generate greater accuracy and easily combined to create an index score of overall gait. Genetic correlations, while difficult to ascertain, are generally low; therefore it is possible to use gait components to select for an overall improvement in both economic traits and gait as part of a balanced breeding programme.
